# Effect of Poly (Sodium 4-Styrene Sulfonate) on the Morphology of Hydroxyapatite Particles

**DOI:** 10.1155/2009/303176

**Published:** 2009-09-16

**Authors:** Nesa Esmaeilian Tari, Mohammad Mahdi Kashani Motlagh

**Affiliations:** Department of Chemistry, Iran University of Science and Technology, Tehran, 16846-13114, Iran

## Abstract

Nanorods hydroxyapatite, (HAP) Ca_10_(PO_4_)_6_(OH)_2_ is successfully prepared by water in oil microemulsion using, CaCL_2_ and H_3_PO_4_ (water phase), poly(sodium 4-styrene sulfonate) (PSSS) as template and cyclohexane as oil phase. The nano-structure of the product was studied by means of X-ray diffraction (XRD), Fourier transmission infrared spectrometer (FT-IR), scanning electron microscopy (SEM), and atomic force microscope (AFM). With this system, we could synthesize nano-particles of hydroxyapatite with high crystallinity and least agglomeration.

## 1. Introduction

Hydroxyapatite (HAP) has been widely studied as an important biocompatible material because of its chemical similarity to the natural calcium phosphate mineral present in a biological hard tissue [1–4]. HAP also finds applications in fields of industrial or technological interests as catalyst in chromatography or gas sensor [5], water purification, fertilizers production, and drug carrier [6]. Properties of HAP, including bioactivity, biocompatibility, solubility, sinterability, castability, fracture toughness. and absorption can be tailored over wide ranges by controlling the particle composition, size, and morphology [7–9].

The morphology of calcium phosphate nanoparticles made by traditional methods as chemical coprecipitation [10], sol-gel [11], spray-pyrolysis [12], hydrothermal synthesis [13], emulsion processing [13], mechano-chemical method [14], and autocombustion methods [15] are needle-like, sheet-like, or spherical which are not more than 300 nm in length. 

Microemulsions are thermodynamically stable dispersions of oil and water stabilized by a surfactant and, in many cases, also a cosurfactant. The microemulsions can be of the droplet type, either with spherical oil droplets dispersed in a continuous medium of water (oil in water microemulsions, O/W) or with spherical water droplets dispersed in continuous medium of oil (water in oil microemulsions, W/O) [16]. 

In our work, we investigated the morphology of nanohydroxyapatite particles formed in the presence of PSSS as a crystal modifier using microemulsion method. The only phase in product as prepared was hydroxyapatite and it was well crystallized.

## 2. Experimental

HAP nanopowders were synthesized using the micelle as a template system where poly(sodium 4-styrene solfonate) (CH_2_CH (C_6_H_4_SO_3_Na), Aldrich) was used as the template. Calcium chloride (CaCl_2_, Merck) and phosphoric acid (H_3_PO_4_ 85%, Merck) were used as calcium and phosphorus sources, respectively. Cyclohexane (Merck) was used as oil phase. For preparing reverse micelle system 10% volume of aqueous solution of 0.02 M polymer was added to cyclohexane, after that aqueous solution of 5 M CaCl_2_ and aqueous solution of 3 M H_3_PO_4_ were added slowly to the reverse micelle, respectively. The mixture was stirring in all steps. Then the pH of microemulsion was adjusted at 12 by adding aqueous solution of NaOH. The final milky suspension was kept for 12 hours at room temperature. The obtained precipitate was then filtered off and washed several times with deionized water. A gel-like paste was produced which was then dried at 150°C for 3 hours and calcined at 650°C for 1 hour.

The morphologies of the as-prepared HAP were observed by a scanning electron microscopy (SEM) (Cambridge-S365) equipped with energy-disperse X-ray spectroscopy and AFM (nanoscope 2). The powder X-ray diffractometer using Cu K*α* (siemens D500) and Fourier transform infrared (FTIR) spectroscopy (shimadzu, KBr pellet technique) was used to identify the quality and composition of hydroxyapatite.

## 3. Results and Discussion

**Figure 1:**
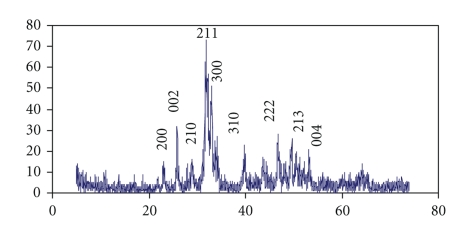
XRD pattern of HAP nanorods.

The wide angle (2*θ* > 10°) X-ray diffraction patterns of the obtained sample is shown in Figure 1. The diffraction peaks correspond to the standard characteristic peaks of hexagonal HAP. There is a high consistency between the data from our sample and that from the standard database, with lattice dimensions of *a* = *b* = 0.9414 nm, *c* = 0.6879 nm (space group p6_3_/m, JCPDS no. 09-0432). No other impurity was observed in the XRD pattern, indicating the chief inorganic phase of the sample is HAP crystal.

**Figure 2:**
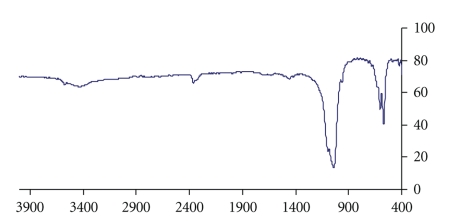
FT-IR spectra of synthesized hydroxyapatite.

Figure 2 shows the FT-IR spectra of the sample. The peak at 3420 cm^−1^ is attributed to the *ν*_2_ bending mode of adsorbed water [17]. The stretching vibration band of OH^−^ is observed at 3569 cm^−1^ [18]. Tow adsorption bands at 561 and 601 cm^−1^ are ascribed to the *ν*_4_ bending mode of PO_4_^3−^ [19]. The characteristic band at 1024 and 1091 cm^−1^ are related to the stretching vibration of PO_4_^3−^. The band at 951 cm^−1^ is assigned to *ν*_1_ stretching mode of PO_4_^3−^. The typical splitting peaks at 567 and 603 cm^−1^ derived from the *ν*_4_ phosphate mode [19]. The FT-IR results indicate that no PSSS molecule is incorporated in the HAP.

**Figure 3:**
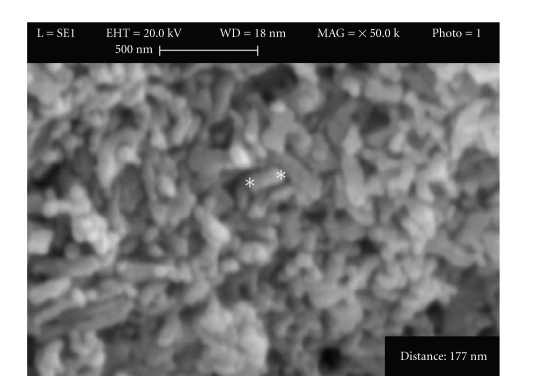
Scanning electron microscopy for synthesized HAP.

Figure 3 shows the SEM image of the HAP nanoparticles. It reveals that the overall morphology of the obtained powders at mentioned situation is rod-like. This suggested that the presence of PSSS had greatly influenced the morphology of the product due to a strong interaction between the sulfate groups of PSSS and the Ca^2+^ ions in the solution and on the surface of HAP particles [20–22].

**Figure 4:**
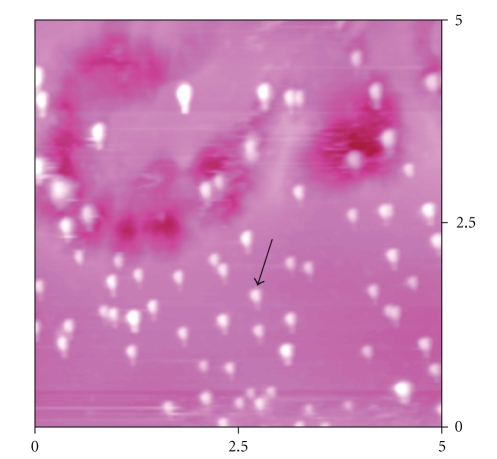
AFM image of hydroxyapatite nanoparticles.

Figure 4 shows the AFM image of nanoparticles of hydroxyaopatite. AFM image confirmed that the particles of synthesized sample are rod shape. AFM image shows the resulted rod shapes HAP have an average width and length about 30 and 200 nm, these sizes are adherent with SEM results which measured the width and length of the shown particle about 40 and 177 nm and also these dimensions evidence that the particles are rod shape. 

We propose here a mechanism for the formation of HAP nanoparticles in the compositions containing the anionic polymer. Earlier studies on PSSS demonstrated that the sulfate groups are able to interact with calcium ions present in an aqueous solution. Surfactant molecules in micelles or emulsion droplet interact with Ca^2+^ ions to form zwitterions structures [20–22]. These numerous calcium-rich domains lead to the fast formation of HAP particles upon contact with phosphate ions in the aqueous phase. The reaction between H_3_PO_4_ and CaCl_2_ in the micelles is deemed to be rapid because of the localized Ca^2+^concentration effect. In addition, the positional stabilization of Ca^2+^ ions within each zwitterions structure as a result of the electrostatic interaction effect by PSSS molecules favors the formation of ordered HAP crystals.

## 4. Conclusion

Drop-wise addition of 0.6 M H_3_PO_4_ solution into 1.0 M CaCl_2_ solution resulted in a fast precipitation of HAP particles. Using PSSS as a nucleation and growth controlling agent, well-crystallized hydroxyapatite nanoparticles were precipitated via a microemulsion route. During the mixing of PSSS with calcium precursor, it formed rod-like micelles which control the morphology and crystallization of nano-hydroxyapatite. X-ray diffraction pattern and FTIR spectrum of the resulted precipitates confirmed the formation of high purity and well-crystallized HAP. The SEM and AFM investigations showed that the obtained HAP nanorods have an average width and length of about 30 and 200 nm, respectively.
